# SUMO-G5C23-D208G@ZIF-F: A Novel Immobilized Enzyme with Enhanced Stability and Reusability for Organophosphorus Hydrolysis

**DOI:** 10.3390/ijms26062469

**Published:** 2025-03-10

**Authors:** Shunye Wang, Ming Ma, Ziyang Wang, Fengqian Cui, Qiqi Li, Zhuang Liu, Dan Wang, Yanan Zhai, Jing Gao

**Affiliations:** 1 School of Pharmacy, Qingdao University, Qingdao 266071, China; 19505420478@163.com; 2 Beijing Institute of Pharmacology and Toxicology, State Key Laboratory of National Security Specially Needed Medicines, Beijing 100850, China; mmdgzyx988@163.com (M.M.); w1352509764@163.com (Z.W.); ya17860396863@163.com (F.C.); 15030578771@163.com (Q.L.); lzzz1027@163.com (Z.L.); wdgs223@163.com (D.W.)

**Keywords:** organophosphorus hydrolase, SUMO fusion expression, ZIF-F, enzyme immobilization, enzyme kinetic parameters

## Abstract

Organophosphorus hydrolase (OPH) is a highly effective bioscavenger for detoxifying hazardous organophosphorus compounds. However, its practical application is hindered by low yield and poor stability. In this study, we employed Small Ubiquitin-like Modifier (SUMO) fusion expression to enhance the solubility of the OPH mutant G5C23-D208G and, for the first time, immobilized the enzyme on a zeolitic imidazolate framework-F (ZIF-F) carrier to improve its stability. The SUMO-G5C23-D208G fusion protein was successfully expressed in *Escherichia coli*, resulting in a yield that was 2.4 times higher than that of native OPH and an 11-fold increase in solubility. The purified protein achieved a purity of 95%. The immobilized enzyme, SU-MO-G5C23-D208G@ZIF-F, exhibited a farfalle-shaped structure with a diameter of approximately 3–5 μm. Compared to the free enzyme, the immobilized enzyme maintained high catalytic efficiency (kcat/Km = 8.9 × 10^4^ M^−1^·s^−1^) and demonstrated enhanced thermal stability, pH stability, and reusability. This study has significantly improved the yield and stability of OPH, thereby supporting its potential for industrial applications.

## 1. Introduction

Organophosphorus has been widely used in pesticides due to its economic cost and broad applications [1]. However, its persistence in the environment and food chain poses health risks. Enzymatic degradation, particularly by OPH, is an efficient and eco-friendly method to mitigate these risks [2]. The enzyme OPH demonstrates lower efficiency in hydrolyzing most organophosphorus compounds compared to its optimal substrate paraoxon, which severely restricts its practical application. However, through directed evolution, researchers have successfully developed a series of mutants with enhanced hydrolytic activity against more organophosphorus. Notably, Cherny et al. [3] constructed a library based on designed sequences and employed a direct measurement method to screen for detoxifying mutants based on OPH. Among these, the G5C23 variant was identified as the most promising candidate, which makes the G5C23 mutant a highly valuable subject for further study in the context of organophosphorus detoxification.

The G5C23 protein produced by traditional techniques tends to accumulate and form inclusion bodies in transformed *Escherichia coli (E. coli)*, resulting in poor solubility and difficulty in further applications. Recent studies have shown that the solubility of the protein can be significantly improved by introducing directed mutations in key residues (such as K185, D208, and R319) [4]. Moreover, the SUMO tag, as an emerging fusion system, has been proven to have significant advantages in enhancing protein solubility and reducing protease degradation. Compared with traditional expression systems, the SUMO tag can act as a molecular chaperone to promote the correct folding of the target protein, while also increasing its stability and solubility [5]. The combination of directed mutation and SUMO tag technology can effectively improve the expression and solubility of the G5C23 protein in *E. coli*, facilitating subsequent research and applications.

Enzymes in practical applications face issues such as poor stability, difficulty in recovery, susceptibility to environmental conditions, and proneness to denaturation and inactivation [6]. In recent years, the method of the in situ immobilization of enzymes using metal–organic frameworks (MOFs) has shown great potential in enhancing enzyme stability [7]. Zeolitic imidazolate frameworks (ZIFs) are a type of MOFs that form tetrahedral topology-based frameworks through coordination between metal ions and imidazolate units [8]. These materials can be synthesized at room temperature and possess excellent stability, making them suitable for enzyme immobilization. The most representative is ZIF-8, which is formed by coordination between metal ions Zn^2+^ and the N atoms on the 2-methylimidazole ring. Chun et al. [9] synthesized a novel butterfly-shaped two-dimensional ZIF, known as ZIF-F, through a dual solvent and zinc source induction method, which shows good thermal stability, solvent stability, and efficient adsorption performance. ZIF-F begins to partially decompose only at 350 °C and exhibits good chemical stability in aqueous solutions with pH values of 1, 2, and 13. It also shows excellent solvent stability in Dimethylformamide, Dichloromethane, and toluene. ZIF-F has an interparticle spacing of 4 nm, a total pore volume of 0.1074 cm^3^·g^−1^, and an adsorption capacity of 182.82 mg·g^−1^. Recent studies have indicated that free enzymes can be embedded in ZIF-F to enhance their stability and potentially increase their activity. Xu et al. [10] employed ZIF-F as a carrier and immobilized papain through in situ synthesis, successfully creating a novel immobilized papain, termed Papain@ZIF-F. Compared to free papain and Papain@ZIF-8, this new immobilized enzyme exhibits significantly higher specific activity and demonstrates excellent reusability.

In this study, G5C23 as a mutant of organophosphorus hydrolase with excellent hydrolytic ability was chosen to be site-directed mutagenized to improve solubility. Additionally, its catalytic efficiency was further enhanced through immobilization technology. We employed a novel metal–organic framework material, ZIF-F, as a carrier and prepared the immobilized enzyme SUMO-G5C23-D208G@ZIF-F via in situ synthesis. Through systematic characterization and activity testing, we evaluated the enzymatic properties of this immobilized enzyme and explored its stability and reusability under various conditions. The findings of this study not only provide a solid foundation for the further application of organophosphorus hydrolases but also offer new insights for their use in the degradation of actual organophosphorus pollutants in the future.

## 2. Results and Discussion

### 2.1. SUMO-G5C23-D208G Expression and Purification

**Figure 1:**
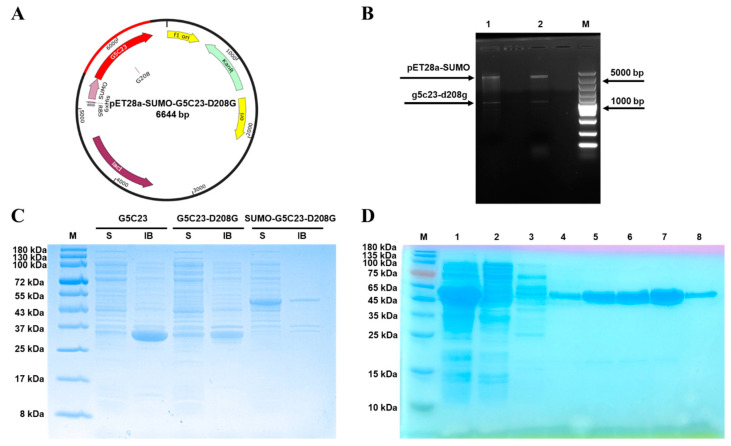
Expression and purification of SUMO-G5C23-D208G. (**A**) Schematic diagram illustrating the construction of the expression vector pET28a-SUMO-G5C23-D208G. (**B**) Double enzymatic digestion (*Bam*H I/*Eco*R I) verification of the recombinant plasmid pET28a-SUMO-G5C23-D208G. M: super DNA marker; 1, 2: double digestion product. (**C**) Protein solubility analysis. The S lane represents soluble cellular lysate; the IB lane represents the inclusion body. (**D**) Sodium dodecyl sulphate–polyacrylamide gel electrophoresis of purification by nickel column. M: protein ladder; 1: centrifugated supernatant; 2: sample effluent; 3: 50 mmol·L^−1^ imidazole eluate; 4: 100 mmol·L^−1^ imidazole eluate; 5: 300 mmol·L^−1^ imidazole eluate of the 1st tube; 6: 300 mmol·L^−1^ imidazole eluate of the 2nd tube; 7: 300 mmol·L^−1^ imidazole eluate of the 3rd tube; 8: 300 mmol·L^−1^ imidazole eluate of the 4th tube.

To improve the soluble expression of the organophosphorus hydrolase mutant G5C23, we initially mutated the aspartic acid (D) at position 208 to glycine (G). This is because aspartic acid (Asp, D) is a negatively charged polar amino acid commonly found on protein surfaces, where it participates in hydrogen bond and salt bridge formation. In contrast, glycine (Gly, G) is the smallest amino acid with a highly flexible side chain, contributing to increased local flexibility and conformational freedom in proteins. The mutation from aspartic acid to glycine can reduce surface charge density and electrostatic repulsion, thereby decreasing the propensity for protein aggregation. Additionally, glycine introduction enhances local flexibility, facilitating the adoption of soluble conformations. This mutation may disrupt specific interactions (e.g., salt bridges or hydrogen bonds) that promote aggregation, ultimately enhancing protein solubility [11,12]. Following this, the synthesized gene encoding the mutant was ligated into the SUMO-tagged cloning vector pET28a(+)-SUMO via enzymatic digestion, resulting in the recombinant expression plasmid pET28a-SUMO-G5C23-D208G (Figure 1A). This plasmid was then introduced into *E. coli* BL21(DE3). The next steps were selecting and picking the monoclonal expression strain, extracting the plasmid, and performing double enzyme digestion verification (Figure 1B). A band of similar size to the target band emerged following double enzyme digestion. A sequencing was performed using the universal primers (T7/T7 ter). The size of pET28a-SUMO is 5633 bp, and the size of the encoded gene sumo-g5c23-d208g is 1377 bp, which is 100% identical to the synthesized gene sequence, proving the successful construction of the recombinant expression strain *E. coli* BL21-SUMO-G5C23-D208G.

The soluble cellular lysate (S) and inclusion body (IB) from induced cultures were analyzed by sodium dodecyl sulfate–polyacrylamide gel electrophoresis (SDS-PAGE) (Figure 1C). As expected, compared to G5C23, there was a significant improvement in the soluble expression levels of G5C23-D208G and SUMO-G5C23-D208G, with SUMO-G5C23-D208G showing the most remarkable increase; the quantitative analysis of protein band grayscale values using ImageJ software (version 1.46r) revealed that the soluble expression level was approximately 11 times higher. The recombinant protein SUMO-G5C23-D208G was purified employing nickel affinity chromatography, with the corresponding results depicted in Figure 1D. According to the literature and the deduced amino acid sequence, the expected molecular weight of the protein is 50 kDa, which is confirmed by the matrix-assisted laser desorption/ionization-time of flight mass spectrometry (MALDI-TOF MS) result showing a monomer molecular weight of 49.9 kDa (Figure S1A), consistent with the predicted value. The non-denaturing gel demonstrated no protein bands between 45–66 kDa but showed a band close to 110 kDa between 66–228 kDa (Figure S1B), indicating that SUMO-G5C23-D208G exists as a dimer. The primary protein band in lanes 5, 6, 7, and 8, which, upon elution with an imidazole buffer at a concentration of 300 mmol·L^−1^, is approximately 55 kDa, aligning with the predicted molecular weight of SUMO-G5C23-D208G. Quantitative analysis revealed that the protein yield of SUMO-G5C23-D208G was 120 mg/L culture, which is 2.4 times higher than the expression level of the wild-type G5C23 reported in the existing literature (50 mg/L culture) [13]. This increase in yield suggests that the solubility and expression of the target protein are significantly enhanced by the specific amino acid substitution at position 208, in conjunction with the SUMO fusion technology. We conducted a preliminary investigation into the properties of the protein. The particle size of SUMO-G5C23-D208G was measured to be 46.68 nm, and its surface charge was −8.856 mV (Figure S2 and Table S2).

### 2.2. Characterization of Immobilized Enzymes

**Figure 2:**
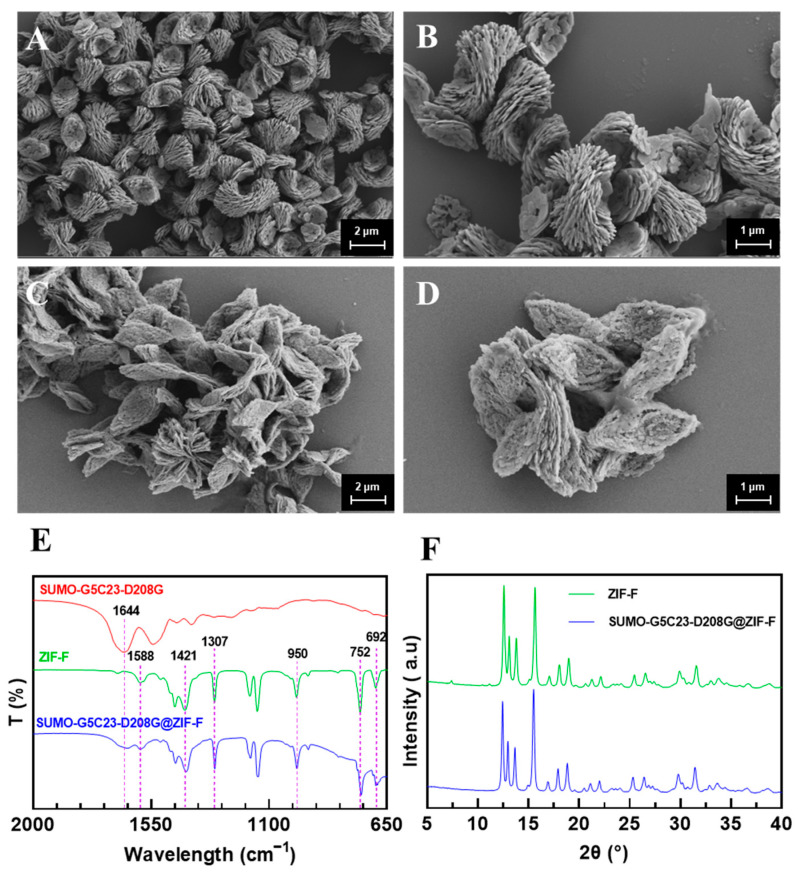
Characterization of immobilized enzymes. Scanning electron microscopic images of ZIF-F at (**A**) 5000× (2 μm bar scale) and (**B**) 10,000× (1 μm bar scale) magnifications. Scanning electron microscopic images of SUMO-G5C23-D208G@ZIF-F at (**C**) 5000× (2 μm bar scale) and (**D**) 10,000× (1 μm bar scale) magnifications. (**E**) The Fourier Transform Infrared Spectroscopy (FT-IR) spectra of SUMO-G5C23-D208G, ZIF-F and SUMO-G5C23-D208G@ZIF-F. (**F**) The X-ray Diffraction (XRD) patterns of the synthesized ZIF-F and SUMO-G5C23-D208G@ZIF-F.

The SUMO-G5C23-D208G was encapsulated in the MOF by the in situ embedding method. Specifically, the mixture containing the zinc acetate solution with SUMO-G5C23-D208G was stirred with an aqueous solution of 2-methylimidazole at room temperature. After 24 h, the synthesized white precipitate (SUMO-G5C23-D208G@ZIF-F) was separated by centrifugation and washed with deionized water. The encapsulation efficiency was found to be 99.9% (the amount of enzyme/protein detected in the supernatant by BCA protein assay was negligible). Figure 2A,B present the Scanning Electron Microscope (SEM) results of ZIF-F, showing that ZIF-F is a farfalle-shaped structure, consists of numerous nanoplates. High-magnification images reveal that the particle size of ZIF-F is approximately 3–5 μm with a good dispersion [9]. Figure 2C,D display the SEM results of SUMO-G5C23-D208G@ZIF-F, indicating that after the encapsulation of the organophosphorus hydrolase SUMO-G5C23-D208G, the particle morphology and size remain essentially unchanged.

As shown in Figure 2E, the out-of-plane bending vibrations of the imidazole ring exhibit strong absorption peaks at 692 cm^−1^ and 752 cm^−1^, whereas the peaks between 900 and 1307 cm^−1^ are assigned as the in-plane bending. The whole ring stretching of imidazole results in the peak at 1421 cm^−1^. The peak at 1588 cm^−1^ is associated with the stretching vibration of C=N [9]. These characteristics indicate that ZIF-F has been successfully synthesized. For SUMO-G5C23-D208G, the amide I band stretching vibration (mainly composed of C=O stretching vibrations) at 1644 cm^−1^ matches the characteristic peak at 1644 cm^−1^ for SUMO-G5C23-D208G@ZIF-F. In the spectrum of ZIF-F without the added enzyme, these characteristic peaks are not present, which preliminarily confirms that the organophosphorus hydrolase has been successfully immobilized on the matrix.

As shown in Figure 2F, SUMO-G5C23-D208G@ZIF-F exhibits strong and sharp characteristic diffraction peaks at 12.5°, 13.0°, 13.5°, and 15.5°, which are completely consistent with the characteristic peaks of ZIF-F. This proves that the addition of the enzyme did not affect the formation of the basic framework of ZIF-F.

### 2.3. Determination of Enzyme Activity and Enzyme Kinetic Parameters

**Figure 3:**
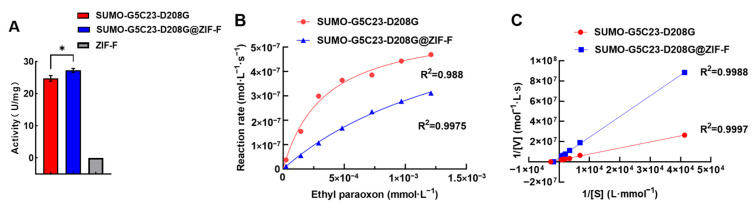
Determination of enzyme activity and kinetic properties. (**A**) Enzyme activity test results. (**B**) Enzyme activity curves. (**C**) Lineweaver–Burk plot. (Mean ± SD, n = 3, * *p* < 0.05).

Using the enzyme activity assay method described, bare ZIF-F did not show the capability to hydrolyze paraoxon. The free organophosphorus hydrolase exhibited an enzyme activity of approximately 25 U/mg. However, after immobilization, as shown in Figure 3A, the specific activity of the enzyme increased, reaching 27 U/mg. This result indicates that, compared to SUMO-G5C23-D208G, the enzyme activity of SUMO-G5C23-D208G@ZIF-F has slightly increased, which may be attributed to the unique structural characteristics of ZIF-F. Specifically, the encapsulation effect of ZIF-F provides the enzyme with a larger specific surface area, and its crystal particles contain more voids and defects (Figure 2D). These characteristics endow the immobilized enzymes with a high specific surface area and low mass transfer resistance, which helps to enhance the enzyme’s activity [14].

The enzyme kinetic curves were plotted based on the experimental results as shown in Figure 3B. The double reciprocal method was used to calculate the enzyme kinetic parameters for SUMO-G5C23-D208G and SUMO-G5C23-D208G@ZIF-F (Figure 3C). The *x*-axis intercept represents the absolute value of 1/*K*_m_, and the *y*-axis intercept represents 1/*V*_max_. The calculations yielded the following parameters for SUMO-G5C23-D208G: *K*_m_ = 3.59 × 10^−4^ M, *V*_max_ = 5.9637 × 10^−7^ M·s^−1^, *k*_cat_ = *V*_max_/total enzyme concentration = 119.034 s^−1^, *k*_cat_/*K*_m_ = 3.3 × 10^5^ M^−1^·s^−1^. For SUMO-G5C23-D208G@ZIF-F, the parameters are as follows: *K*_m_ = 1.71 × 10^−3^ M, *V*_max_ = 7.635 × 10^−7^ M·s^−1^, *k*_cat_ = *V*_max_/total enzyme concentration = 129.5 s^−1^, *k*_cat_/*K*_m_ = 8.9 × 10^4^ M^−1^·s^−1^. These parameters indicate that SUMO-G5C23-D208G@ZIF-F has a reduced affinity for the substrate compared to the free SUMO-G5C23-D208G, possibly due to some distortion or deformation of the enzyme’s active site during the immobilization process, which altered the three-dimensional structure of the active site and weakened the enzyme’s binding capacity to the substrate. However, compared to the free enzyme, the *k*_cat_ has improved and the results of enzyme kinetics analysis also confirmed the phenomenon of the increased activity of the immobilized enzymes. Although the *K*_m_ value of the immobilized enzyme was slightly higher, the increase in its *k*_cat_ value suggests that the mass transfer efficiency of the substrate in the immobilized enzyme system may be higher, which compensates for the slight decrease in substrate affinity, thereby leading to a slight increase in enzyme activity [15]. The final similar *k*_cat_/*K*_m_ values demonstrate that the immobilized enzyme maintains good catalytic efficiency towards the substrate ethyl paraoxon.

### 2.4. Examination of the Enzymatic Properties

#### 2.4.1. Catalytic Performance of Biocatalysts

**Figure 4:**
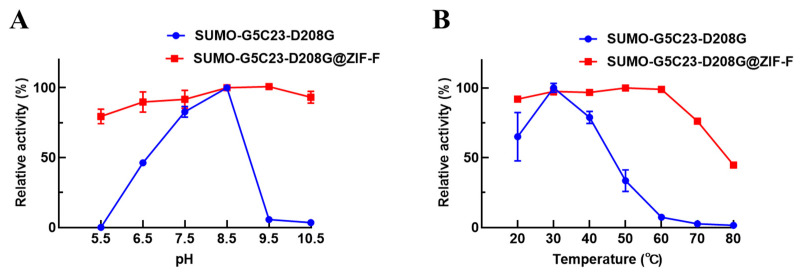
pH (**A**) and temperature (**B**) on the activities of free and immobilized SUMO-G5C23-D208G. (Mean ± SD, n = 3).

The effects of pH on the activity of the free and immobilized SUMO-G5C23-D208G are shown in Figure 4A. The free enzyme reaches its maximum activity at a pH value of 8.5. In contrast, SUMO-G5C23-D208G@ZIF-F exhibits relatively high relative activity over a pH range of 5.5 to 10.5, with the most suitable pH value being 9.5. This indicates that the immobilized SUMO-G5C23-D208G maintains high enzyme activity over a broader pH range. The structure of ZIF-F does not undergo significant changes in the pH range of 5.5–10.5 (Figure S3A–G). Furthermore, the effects of temperature on the activity of the free and immobilized SUMO-G5C23-D208G are shown in Figure 4B; SUMO-G5C23-D208G exhibits its highest activity at 30 °C. In contrast, SUMO-G5C23-D208G@ZIF-F displays the highest enzymatic activity at 50 °C. The optimal temperature for SUMO-G5C23-D208G@ZIF-F is higher than that for the free SUMO-G5C23-D208G, which may be attributed to the restricted domain effect of the immobilized enzyme molecules, requiring higher activation energy to exhibit activity [16]. With further increase in temperature, both the free and immobilized SUMO-G5C23-D208G show varying degrees of activity decline. The observed phenomena can be ascribed to the irreversible alterations in the conformation of enzyme molecules when subjected to elevated temperatures, which result in a consequent decline in their catalytic functionality [17]. It is noteworthy that the immobilized SUMO-G5C23-D208G maintains relatively stable enzymatic activity within the temperature range of 20–60 °C. This suggests that the SUMO-G5C23-D208G immobilized within the pores of ZIF-F provides a favorable microenvironment for the enzyme, thereby protecting the active site and exhibiting more stable activity [18].

#### 2.4.2. Analysis of Stability Performance

**Figure 5:**
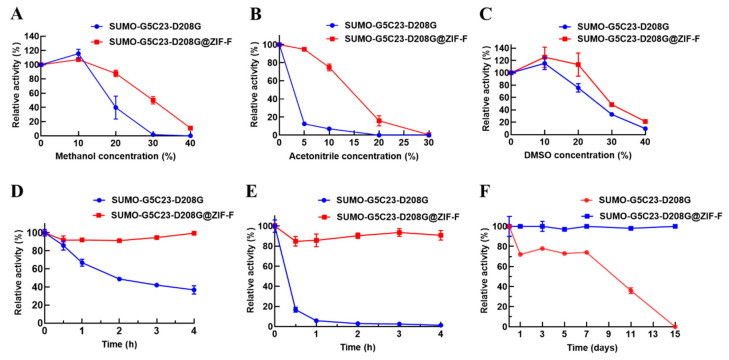
Stability performance analysis of SUMO-G5C23-D208G and SUMO-G5C23-D208G@ZIF-F with various organic solvents or different temperature environments: (**A**) methanol; (**B**) acetonitrile; (**C**) DMSO. Thermal stability results at (**D**) 40 °C and (**E**) 50 °C. (**F**) Storage stability of immobilized enzymes at 25 °C. (Mean ± SD, n = 3).

Organophosphorus hydrolase can effectively degrade organophosphorus pesticides. However, this process typically requires the use of polar organic solvents for dissolution, which may lead to the loss of enzymatic activity [19]. Therefore, this study selected methanol, acetonitrile, and dimethyl sulfoxide (DMSO) to test the organic solvent tolerance of free SUMO-G5C23-D208G and SUMO-G5C23-D208G@ZIF-F. The results, as shown in Figure 5A–C, indicate that organic solvents significantly affect the enzymatic activity of free enzymes, with high concentrations of organic solvents reducing enzymatic activity, while immobilized enzymes exhibit a mitigated impact. Notably, within a volume fraction concentration ≤10%, methanol has an activating effect on both free SUMO-G5C23-D208G and SUMO-G5C23-D208G@ZIF-F. Above a concentration of 10%, the effect of methanol on the enzyme rapidly transitions to an inhibitory effect. Similarly, within a volume fraction concentration ≤20%, DMSO has an activating effect on both free SUMO-G5C23-D208G and SUMO-G5C23-D208G@ZIF-F. Above a concentration of 10%, the effect of DMSO on the enzyme transitions to an inhibitory effect. The reason may be that when the reaction system contains methanol or DMSO, it affects the interactions between the side chains of enzyme molecules or between enzyme molecules and water, leading to minor changes in the forces that maintain the protein molecular conformation. These changes may increase the flexibility of the enzyme’s active site, which is favorable for the function of the enzyme, thus exhibiting an activating effect on the enzyme [20]. As the content of DMSO and methanol increases, it further deprives the enzyme of its hydration layer, causing the hydrogen bonds, hydrophobic bonds, and van der Waals forces formed directly or indirectly by water molecules to be disrupted, leading to drastic changes in the conformation of the enzyme’s active center and a sharp decrease in enzymatic activity [21]. This is consistent with the concept that enzymes often appear more “rigid” in organic solvents due to the lack of water molecules that act as a “molecular lubricant” in aqueous media, facilitating reversible conformational changes during substrate binding [22]. In summary, the initial activating effect of methanol and DMSO at low concentrations can be attributed to the increased flexibility of the enzyme’s active site, while the subsequent inhibitory effect at higher concentrations is due to the disruption of the enzyme’s hydration layer and the resulting conformational rigidity.

To investigate the thermal stability of free SUMO-G5C23-D208G and SUMO-G5C23-D208G@ZIF-F, both free and immobilized enzymes were incubated at 40 and 50 °C for a period of time, after which their residual enzyme activities were studied. As shown in Figure 5D,E, after incubation at 40 °C and 50 °C for 4 h, SUMO-G5C23-D208G@ZIF-F can maintain more than 95% of its initial enzymatic activity, while free SUMO-G5C23-D208G can only maintain 40% and even 0% of its initial activity under the same conditions, respectively. The results indicate that immobilized enzymes have better stability than free enzymes under high-temperature conditions. SUMO-G5C23-D208G@ZIF-F can resist conformational changes in enzyme molecules caused by high temperatures, which may be due to the electrostatic interactions between SUMO-G5C23-D208G enzyme molecules and the ZIF-F carrier. These interactions allow the enzyme molecules to maintain their original conformation for extended periods at high temperatures, thereby maintaining higher enzymatic activity [23].

The storage stability of free SUMO-G5C23-D208G and SUMO-G5C23-D208G@ZIF-F was studied. As shown in Figure 5F, under ambient conditions, the activity of the ZIF-F immobilized enzyme remained essentially unchanged over 15 days, while the activity of the free enzyme was almost completely lost within the same period. This indicates that enzyme immobilization overcomes the limitations of free enzyme storage at room temperature, enhancing chemical stability and environmental tolerance [24].

### 2.5. Reusability Examination of Immobilized Enzyme

**Figure 6:**
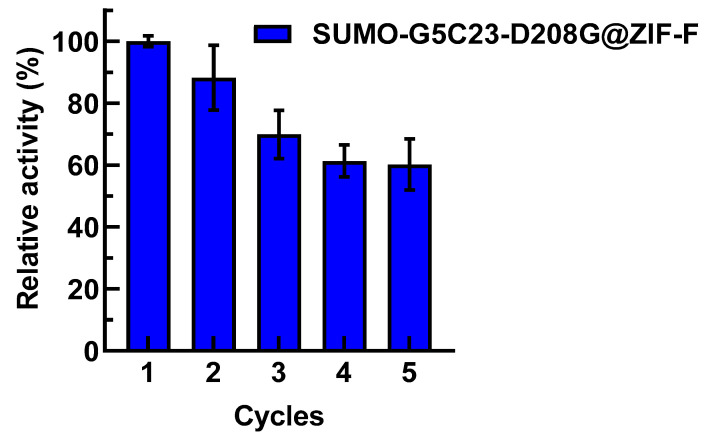
Reusability results of the immobilized enzyme. (Mean ± SD, n = 3).

Due to the convenience of recovery and separation for the reuse of immobilized enzymes, which can effectively reduce the cost of enzyme use, they have attracted widespread attention. We have conducted a study on the reusability of SUMO-G5C23-D208G@ZIF-F. As shown in Figure 6, after being reused five times, the relative activity of SUMO-G5C23-D208G@ZIF-F decreased to 60%, which may be due to the loss of immobilized enzyme during the centrifugation and washing processes [15]. However, the fact that the enzyme still retains 60% of its activity after five reuses demonstrates the good reusability of SUMO-G5C23-D208G@ZIF-F.

## 3. Materials and Methods

### 3.1. Materials

Ethyl paraoxon (purity ≥ 98%) was purchased from Beijing Wanjia Shouhua Biological Co., Ltd. (Beijing, China); HEPES buffer solution was purchased from Beijing Leigen Biotechnology Co., Ltd. (Beijing, China); yeast extract, peptone was purchased from UK OXOID Company (Basingstoke, UK); *p*-Nitrophenol was purchased from USA Sigma-Aldrich Company (St. Louis, MO, USA); imidazole, 12% SDS-PAGE rapid preparation kit, 10×SDS-PAGE electrophoresis buffer, ExBlue protein ultra-fast staining solution and colored pre-stained protein molecular weight standard were purchased from Beijing Zhuangmeng International Bio-gene Technology Co., Ltd. (Beijing, China); 2-Methylimidazole, zinc acetate dihydrate were purchased from Shanghai Macklin Biochemical Technology Co., Ltd. (Shanghai, China); Super DNA Marker was purchased from Kangwei Century Biotechnology Co., Ltd. (Beijing, China); kanamycin sulfate, isopropyl β-D-thiogalactopyranoside (IPTG) were purchased from Shanghai Yisheng Biotechnology Co., Ltd. (Shanghai, China). Plasmid cloning host strain *Escherichia coli* DH5α, protein expression host strain *E. coli* BL21(DE3) were purchased from Kangwei Century Biotechnology Co., Ltd. (Beijing, China); protein expression plasmid pET28a(+) was purchased from Beijing Zhuangmeng International Bio-gene Technology Co., Ltd. (Beijing, China); DNA restriction endonucleases *Eco*R I (FD0274) and *Bam*H I (FD0054) were purchased from USA Thermo Fisher Scientific Company (Waltham, MA, USA).

### 3.2. Construction of Expression Vector for the Organophosphorus Hydrolase Mutant SUMO-G5C23-D208G

Based on the reported encoding gene of the organophosphorus hydrolase mutant G5C23 from the literature [3], the 208th amino acid of G5C23 was mutated from aspartic acid (D) to glycine (G). The corresponding encoded gene was named *g5c23-d208g* and underwent *E. coli* codon optimization and full gene synthesis. The synthesized gene was connected to the SUMO-tagged [25] cloning vector pET28a(+)-SUMO via the restriction sites *Bam*H I and *Eco*R I, resulting in the recombinant expression plasmid pET28a-SUMO-G5C23-D208G. The gene synthesis service was provided by Nanjing GenScript Biotech Co., Ltd. (Nanjing, China) (Table S1).

### 3.3. Construction of Expression Strain for the Organophosphorus Hydrolase Mutant SUMO-G5C23-D208G

Add 10 μL of the recombinant expression plasmid to approximately 100 μL of *E. coli* DH5α competent cells, gently mix, and place on ice for 30 min. Apply heat shock at 42 °C for 90 s, then place on ice for 2 min. Add 500 μL of LB liquid medium and cultivate at 37 °C with a shaking speed of 220 r·min^−1^ for 45 min. Spread an appropriate amount of bacterial liquid onto LB plates (containing 2% agar) with kanamycin sulfate (Kan, 100 μg·mL^−1^) and incubate at 37 °C for 18 h. Pick single colonies and extract plasmids for double enzyme digestion verification, and screen positive clones for further sequencing verification. Extract plasmids from the correct strains verified by sequencing, and transform *E. coli* BL21(DE3) competent cells using the same method mentioned above; pick single colonies, extract plasmids for sequencing verification, and obtain the expression strain [26].

### 3.4. Fermentation of Bacterial Cells

The SUMO-G5C23-D208G expression strain was revived in LB medium containing kanamycin sulfate (50 μg·mL^−1^) at 37 °C with a shaking speed of 200 r·min^−1^ for 16 h. It was then inoculated into TB medium containing kanamycin sulfate (25 μg·mL^−1^) at a seeding volume of one-tenth of the original volume, and cultivated at 37 °C with a shaking speed of 180 r·min^−1^ for 5 h until the OD600 reached approximately 0.6. The inducer Isopropyl β-D-1-thiogalactopyranoside (IPTG) at a concentration of 0.25 mmol·L^−1^ was added, and the culture was continued at 25 °C with a shaking speed of 180 r·min^−1^ for 24 h. The cells were then centrifuged at 10,000 r·min^−1^ for 3 min, the supernatant was discarded, and the pellet was weighed and stored at −80 °C in a refrigerator [26].

### 3.5. Purification and Characterization of Organophosphorus Hydrolase

#### 3.5.1. Preparation of Crude Organophosphorus Hydrolase and Ni-Column Purification

Retrieve the bacterial cells stored at −80 °C, and add 100 mL of buffer containing 10 mM imidazole, 20 mM 4-(2-hydroxyethyl) piperazine-1-ethanesulfonic acid (HEPES), and 0.5 M sodium chloride (pH = 8) for every 10 g of cells, and resuspend completely at room temperature. Sonicate the cells in an ice bath using an ultrasonic disruptor with a 12 mm probe. The sonication conditions are as follows: 75% power, 2 s on, 2 s off, for a total of 40 min. Replace the ice bath after 20 min of sonication. The lysates were then partitioned into soluble and insoluble fractions by centrifugation at 10000 r·min^−1^ for 30 min at 4 °C. The proteins were separated and detected by SDS–PAGE. Collect the supernatant, filter through qualitative filter paper, and keep it on ice for later use. Use a nickel column (Cytiva, HisTrapTM HP) with a flow rate of 5 mL·min^−1^ and a maximum column pressure of 0.2 Mpa. First, rinse the column with 5 times the column volume of deionized water, then equilibrate with 5 times the column volume of buffer containing 10 mM imidazole, 20 mM HEPES, and 0.5 M sodium chloride (pH = 8). Load the sample on ice, and after loading, equilibrate with 5 times the column volume of the same buffer. Elute the impurities using buffers containing 50 mM imidazole and 100 mM imidazole, respectively, until the impurity peaks are completely eluted (the baseline becomes flat). Elute the target protein peak with a buffer containing 300 mM imidazole, 20 mM HEPES, and 0.5 M sodium chloride (pH = 8) for 5 times the column volume, collect the fractions containing the target protein peak, and verify the purification effect using SDS-PAGE [26].

#### 3.5.2. SDS-PAGE Verification of Fractions After Ni-Column Purification

Prepare a 1 mm gel according to the instructions of the 12% SDS-PAGE rapid preparation kit. Take 20 μL of the flow-through and different concentration eluates, add 5 μL of loading buffer, and boil in water for 10 min. After cooling to room temperature, take 20 μL for SDS-PAGE. The electrophoresis conditions are 120 V for 85 min. After electrophoresis, stain with Coomassie Brilliant Blue staining solution, wash with deionized water, and then photograph [26].

### 3.6. Preparation of Immobilized Enzymes

#### 3.6.1. Synthesis of ZIF-F

Accurately weigh 270 mg (1.2 mmol) of zinc acetate and dissolve it in 15 mL of deionized water to form solution a. Similarly, accurately weigh 410 mg (5.0 mmol) of 2-methylimidazole and dissolve it in 15 mL of deionized water to form solution b. Then, mix solution b with solution a and continuously stir at a rate of 300 r·min^−1^ for 24 h to promote the reaction. After the reaction is complete, the resulting mixture is centrifuged at 10,000 r·min^−1^ for 10 min, discard the supernatant, and wash the precipitate three times with deionized water to remove any unreacted raw materials and by-products, ultimately obtaining pure ZIF-F product [10].

#### 3.6.2. Synthesis of SUMO-G5C23-D208G@ZIF-F

Accurately weigh 270 mg (1.2 mmol) of zinc acetate and dissolve it in 10 mL of deionized water. Then, add 5 mL of a 0.5 mg·mL^−1^ SUMO-G5C23-D208G solution (dissolved in 0.1 mol·L^−1^ pH 7.4 Tris-HCl buffer) to this solution. Label the resulting mixed solution as solution a. Next, dissolve 410 mg (5.0 mmol) of 2-methylimidazole in 15 mL of deionized water to form solution b. Mix solution b with solution a and continuously stir at a rate of 300 r·min^−1^ for 24 h. After stirring is complete, the mixture is centrifuged at 10,000 r·min^−1^ for 10 min, the supernatant is collected, and the concentration of free enzymes that were not encapsulated in the immobilized enzyme is measured using the BCA protein concentration assay. The immobilization efficiency of the enzyme is calculated using the formula: Yield (%) = 100 × (immobilized activity/starting activity) [27]. The precipitate is washed three times with deionized water to remove unreacted raw materials and by-products. The final product obtained is SUMO-G5C23-D208G@ZIF-F [10].

### 3.7. Analysis of Immobilized Enzyme Features

#### 3.7.1. SEM Characterization of Immobilized Enzyme Morphology

Cut a silicon wafer into approximately 4 mm × 4 mm squares using a glass cutter. Use a pipette to 5 μL of diluted sample and drop it onto the silicon wafer, allowing it to air dry naturally. Then, fix the silicon wafer to an aluminum sample stage with conductive adhesive and perform scanning electron microscopy testing with JSM-7900F (JEOL Ltd., Tokyo, Japan), observing the material morphology at different magnifications [28].

#### 3.7.2. FT-IR

Place a small amount of powder sample in a mortar, add 0.2 g of dry potassium bromide, grind and mix evenly, then press into a pellet. Use a Nicolet iS20 scanning (Thermo Fisher Scientific, MA, USA) infrared spectrometer to test the infrared spectrum from 650 to 2000 cm^−1^ [29].

#### 3.7.3. XRD

Cut a glass slide into a square with a side length greater than 1 cm using a glass cutter. After centrifuging the sample and discarding the supernatant, add 300 μL of water to fully dissolve it. Use a pipette to drop the solution onto the glass slide. Conduct X-ray diffraction tests with Rigaku SmartLab SE (Rigaku Corporation, Tokyo, Japan), using Cu Kα radiation, λ = 0.15418 nm, with an applied voltage of 40 kV, current of 30 mA, and 2θ ranging from 5 to 60°.

### 3.8. Evaluation of Enzyme Activity and Kinetic Properties

#### 3.8.1. Enzyme Activity Assay

Organophosphorus hydrolase can hydrolyze its substrate paraoxon into *p*-nitrophenol. One molecule of paraoxon can generate one molecule of *p*-nitrophenol. Therefore, the activity of the samples can be compared by measuring the amount of *p*-nitrophenol generated under the same conditions. Accurately weigh 20.86 mg (6 mmol) of *p*-nitrophenol in a beaker, add 5 mL of deionized water to dissolve it, and make up to 25 mL in a volumetric flask to obtain a 6 mM *p*-nitrophenol solution. Prepare standard solutions of different concentrations of *p*-nitrophenol, take 200 μL of each, and measure the OD value at 405 nm to plot the standard curve of *p*-nitrophenol. Using the BCA method to determine the protein concentration of each sample, dilute with pH = 8.0 50 mM HEPES to a concentration of 5 μg·mL^−1^. Take 4 × 5 mL centrifuge tubes, set tube 1 as a blank control, and tubes 2, 3, and 4 as the SUMO-G5C23-D208G experimental group. Add 5 μL of 10 mg·mL^−1^ (0.0483 mol/L) ethyl paraoxon solution and 900 μL of 50 mM pH 8.0 HEPES buffer to the centrifuge tubes. Add 1 mL of 10% trichloroacetic acid to tube 1, then add 100 μL of SUMO-G5C23-D208G solution (10% trichloroacetic acid can inactivate the enzyme and terminate the reaction), place in 37 °C; then add 100 μL of SUMO-G5C23-D208G solution to tubes 2, 3, and 4 at intervals of 30 s. After 10 min, remove tube 1, then remove tubes 2, 3, and 4 at intervals of 30 s and immediately add 10% trichloroacetic acid. Add 1 mL of 10% sodium carbonate solution to each of the 4 centrifuge tubes for color development, mix well, and then take out 200 μL of each sample to measure the absorbance at 405 nm using a plate reader. Each sample is measured in triplicate, and the enzyme activity (U/mg) = the amount of *p*-nitrophenol (μM) × (1 mg/0.1 mL· sample concentration)/10 min. When the absorbance of the sample is substituted into the standard curve, the absorbance of the blank control needs to be subtracted. Take another 4 × 5 mL centrifuge tubes and measure the activity of SUMO-G5C23-D208G@ZIF-F using the same method as for SUMO-G5C23-D208G [26]. Paraoxon is a highly toxic chemical, this substance is known for its acute toxicity, posing significant risks to human health and safety. Exposure to paraoxon, even in small quantities, can lead to severe symptoms such as respiratory distress, convulsions, and central nervous system depression. In extreme cases, it may result in irreversible damage or even death. Given these alarming potential consequences, it is essential to approach any work involving paraoxon with the utmost caution and strict adherence to safety protocols. To ensure the safety of the experiment, we implemented the following measures: All researchers handling paraoxon were required to wear appropriate personal protective equipment (PPE), including lab coats, gloves, and goggles. We had emergency response equipment, such as eyewash stations and safety showers, readily available in the laboratory. The experiments were conducted in a well-ventilated fume hood to minimize exposure to toxic vapors. Any waste containing paraoxon was disposed of in accordance with local regulations to prevent environmental contamination.

#### 3.8.2. Investigation of Enzyme Kinetic Parameters

Take 200 μL of a 10 mg·mL^−1^ (36.3 mM) solution of paraoxon and dilute it with 800 μL of deionized water to a final volume of 1 mL to prepare a 2 mg·mL^−1^ paraoxon solution. The reaction and detection conditions are the same as the method used for the enzyme activity assay: measure the amount of *p*-nitrophenol produced and calculate the enzyme kinetic constants *K*_m_, *V*_max_, *k*_cat_, and *k*_cat_/*K*_m_ using the Michaelis–Menten equation. The specific procedure is as follows: Take 1, 6, 8, 12, 20, 30, 40, and 50 μL from a 2 mg·mL^−1^ (0.00965 mol/L) paraoxon solution and add them to 1.5 mL centrifuge tubes, bringing the volume up to 150 μL with pH 8.0 50 mM HEPES, and label them in sequence. Add 150 μL of the sample (SUMO-G5C23-D208G at a concentration of 500 ng·mL^−1^ and SUMO-G5C23-D208G@ZIF-F at a concentration of 500 ng·mL^−1^), immediately place the test tube into 37 °C, and time each tube for 10 min. Afterward, add 300 μL of 10% trichloroacetic acid solution to terminate the reaction, and then add 300 μL of 10% sodium carbonate solution for color development. The amount of *p*-nitrophenol generated per unit time is the reaction rate [26].

### 3.9. Examination of Enzymatic Properties of SUMO-G5C23-D208G and SUMO-G5C23-D208G@ZIF-F

#### 3.9.1. Optimal pH Examination

Immerse the free enzymes and immobilized enzymes separately in reaction buffers of different pH values. The specific buffers are 50 mM HEPES buffers with various pH values as 5.5, 6.5, 7.5, 8.5, 9.5, and 10.5. The reaction and detection conditions are the same as the method used for the enzyme activity assay. Define the highest enzyme activity as 100%, calculate the relative enzyme activity, and determine the pH value corresponding to the highest enzyme activity as the optimal pH value.

#### 3.9.2. Optimal Temperature Examination

Resuspend the free enzymes and immobilized enzymes separately in HEPES buffer (50 mM, pH = 8.0) and conduct the enzyme reaction at temperatures of 20, 30, 40, 45, 50, 55, 60, 70, and 80 °C. Measure the enzyme activity at each temperature. The reaction and detection conditions are the same as the method used for the enzyme activity assay. Define the highest enzyme activity as 100%, calculate the relative enzyme activity, and determine the temperature corresponding to the highest enzyme activity as the optimal temperature.

#### 3.9.3. Organic Solvent Tolerance Examination

To study the effect of organic solvents on free and immobilized enzymes, disperse SUMO-G5C23-D208G and SUMO-G5C23-D208G@ZIF-F with corresponding protein concentrations in different concentrations of organic solvents (methanol, acetonitrile, dimethyl sulfoxide) and react for 10 min. The reaction and detection conditions are the same as the method used for the enzyme activity assay, defining the highest enzyme activity as 100%.

#### 3.9.4. Thermal Stability Examination

To investigate the thermal stability of free and immobilized enzymes, place a certain amount of SUMO-G5C23-D208G and SUMO-G5C23-D208G@ZIF-F in HEPES buffer (50 mM, pH = 8.0) and incubate them at 40 °C and 50 °C water-bath conditions. Measure their remaining activity at 30 min, 1 h, 2 h, 3 h, and 4 h. The reaction and detection conditions are the same as the method used for the enzyme activity assay, defining the highest enzyme activity as 100%.

#### 3.9.5. Storage Stability Examination

Place a certain amount of SUMO-G5C23-D208G and SUMO-G5C23-D208G@ZIF-F in a 25 °C incubator and measure their remaining activity on days 0, 1, 3, 5, 7, 11, and 15. The reaction and detection conditions are the same as the method used for the enzyme activity assay.

#### 3.9.6. Examination of the Reusability of Immobilized Enzyme

Take a certain amount of immobilized enzymes and react them with a substrate solution of paraoxon at a concentration of 10 mg·mL^−1^ for 10 min. Then, centrifuge and measure the absorbance of the supernatant at 405 nm. Wash the immobilized enzymes obtained after the catalytic reaction with deionized water three times and reuse them for the next reaction. Repeat the above steps for 5 times. Define the specific activity of SUMO-G5C23-D208G@ZIF-F in the first reaction as 100% and calculate the relative enzyme activity.

## 4. Conclusions

The current study successfully utilized site-directed mutagenesis and SUMO fusion technology to enhance the solubility and expression of the organophosphorus hydrolase mutant G5C23-D208G. The soluble expression was improved by approximately 11-fold, and the yield of the recombinant protein was significantly increased by 2.4 times compared to the wild-type, thereby demonstrating the efficacy of our approach in promoting the soluble expression of the target enzyme.

The immobilization of the enzyme on the metal–organic framework ZIF-F resulted in a biocatalyst with enhanced thermal, pH stability, and reusability. The immobilized SUMO-G5C23-D208G@ZIF-F exhibited a similar catalytic efficiency (*k*_cat_/*K*_m_) to the free enzyme, indicating that the immobilization process did not compromise the enzyme’s ability to catalyze the hydrolysis of organophosphorus compounds. The improved stability and reusability of the immobilized enzyme make it a promising candidate for industrial applications, particularly in the detoxification of organophosphorus pesticides and nerve agents.

The results of this study provide a solid foundation for the further optimization of the immobilization process and exploration of the potential applications of SUMO-G5C23-D208G@ZIF-F in the degradation of actual organophosphorus pollutants. Future work will focus on scaling up the production of the immobilized enzyme and evaluating its performance in real-world scenarios, thereby contributing to the development of sustainable and effective solutions for environmental remediation and human health protection.

## Data Availability

Data is contained within the article and Supplementary Materials.

## Supplementary Materials

The following supporting information can be downloaded at: https://www.mdpi.com/article/10.3390/ijms26062469/s1.

## Abbreviations

The following abbreviations are used in this manuscript:

OPH	organophosphorus hydrolase
SUMO	Small Ubiquitin-like Modifier
ZIF-F	zeolitic imidazolate framework-F
MOFs	metal–organic frameworks
SDS-PAGE	sodium dodecyl sulfate–polyacrylamide gel electrophoresis
SEM	Scanning Electron Microscope
FT-IR	Fourier Transform Infrared Spectroscopy
XRD	X-ray Diffraction
