# Supplementary material for: SUMO-G5C23-D208G@ZIF-F: A Novel Immobilized Enzyme with Enhanced Stability and Reusability for Organophosphorus Hydrolysis
Source: Int J Mol Sci. 2025 Mar 10;26(6):2469. doi: 10.3390/ijms26062469 (PMC11942619; doi:10.3390/ijms26062469)
Supplement: Supplementary file 1 [file ijms-26-02469-s001.zip › ijms-3480394-supplementary.pdf]

Supplementary Material

SUMO-G5C23-D208G@ZIF-F: A Novel Immobilized Enzyme with Enhanced Stability and Reusability for Organophosphorus Hydrolysis

Shunye Wang [1, 2], Ming Ma [2], Ziyang Wang [2], Fengqian Cui [2], Qiqi Li [2], Zhuang Liu [2], Dan Wang [2], Yanan Zhai [2], [*] and Jing Gao [2*]

[1]School of Pharmacy, Qingdao University, Qingdao, Shandong 266071, China.

[2]Beijing Institute of Pharmacology and Toxicology, State Key Laboratory of National Security Specially Needed Medicines, Beijing, 100850, China.

[*]Correspondence: Jing Gao, Beijing Institute of Pharmacology and Toxicology, Email gjsmmu@126.com; Yanan Zhai, Beijing Institute of Pharmacology and Toxicology, Email zyn1989@mail.ustc.edu.cn

Contents

Supplementary Figures

Figure S1. Protein molecular weight characterization results

Figure S2. Investigation of protein properties

Figure S3. The impact of different pH values on the structure of ZIF-F

Supplementary Tables

Table S1. Protein sequences used in this study

Table S2. Particle size and Zeta potential statistics of SUMO-G5C23-D208G

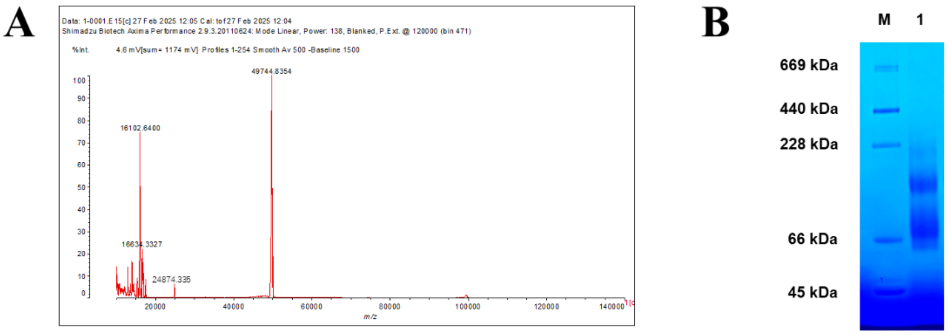

Figure S1. Protein molecular weight characterization results (A) MALDI-TOF mass spectrometry analysis results. (B) Non-denaturing gel electrophoresis analysis. Lane M represents the molecular weight marker, and lane 1 contains the SUMO-G5C23-D208G protein sample, which shows the dimeric state of the protein.

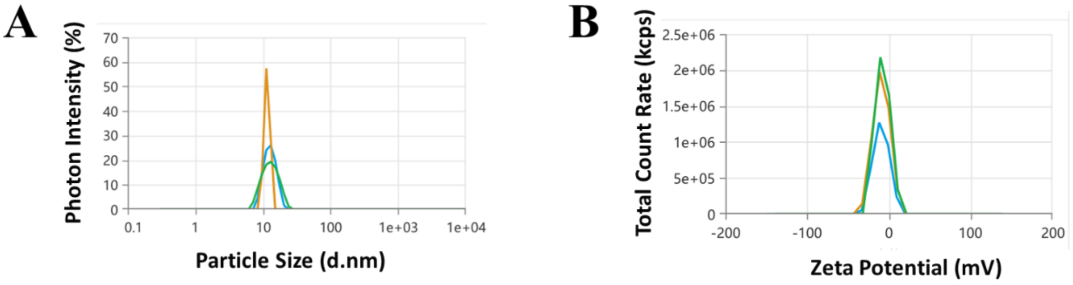

Figure S2. Investigation of protein properties (A) Particle size. (B) Zeta potential

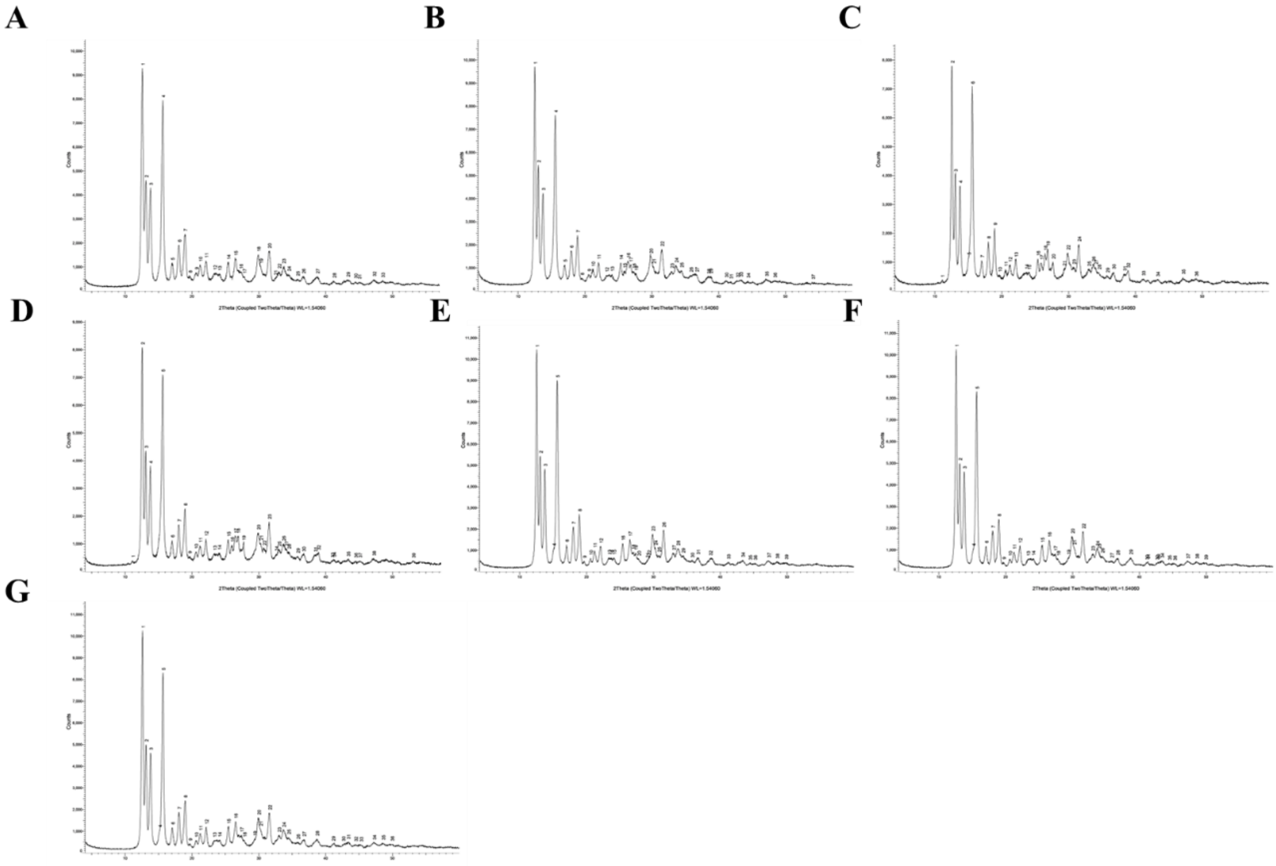

Figure S3. The impact of different pH values on the structure of ZIF-F (A) Untreated ZIF-F (B) pH=5.5; (C) pH=6.5; (D) pH=7.5; (E) pH=8.5; (F) pH=9.5; (G) pH=10.5.

Table S1. Protein sequences used in this study

Protein	Squence
G5C23-D208G	MASIGTGDRINTVRGPITISEAGFTLTHEHICGSSAGFLRAWPE
	FFGSRAALVEKAVRGLRRARAAGVRTIVDVSTFDIGRDVSLL
	AEVSRAADVHIVAATGLWEDPPLSMRLRSVEELTQFFLREIQY
	GIEDTGIRAGIIKVATNGKATPFQELVLKAAARASLATGVPVT
	THTAASQRGGEQQAAIFESEGLSPSRVCIGHSDDTDDLSYLTA
	LAARGYLIGLDGIPHSAIGLEDNASASALLGNRSWQTRALLI
	KALIDQGYMKQILVSNDWLFGFSSYVTNIMDVMDRVNPDG
	MAFIPLRVIPFLREKGVPQETLAGITVTNPARFLSPTLRAS
SUMO-G5C23-D208G	MGSSHHHHHHSSGLVPRGSHMASMSDSEVNQEAKPEVKPEV
	KPETHINLKVSDGSSEIFFKIKKTTPLRRLMEAFAKRQGKEMD
	SLRFLYDGIRIQADQTPEDLDMEDNDIIEAHREQIGGSSIGTGD
	RINTVRGPITISEAGFTLTHEHICGSSAGFLRAWPEFFGSRAAL
	VEKAVRGLRRARAAGVRTIVDVSTFDIGRDVSLLAEVSRAAD
	VHIVAATGLWEDPPLSMRLRSVEELTQFFLREIQYGIEDTGIRA
	GIIKVATNGKATPFQELVLKAAARASLATGVPVTTHTAASQRG

GEQQAAIFESEGLSPSRVCIGHSDDTDDLSYLTALAARGYLIG
LDGIPHSAIGLEDNASASALLGNRSWQTRALLIKALIDQGYM
KQILVSNDWLFGFSSYVTNIMDVMDRVNPDGMAFIPLRVIPF
LREKGVPQETLAGITVTNPARFLSPTLRAS

Table S2. Particle size and Zeta potential statistics of SUMO-G5C23-D208G

Name	Average	SD	RSD
Z-Average (nm)	46.68	1.8290	0.039
Polydispersity Index (PDI)	0.1709	0.1709	0.1709
Zeta Potential (mV)	-8.856	0.9307	10.51
